# Herd diagnosis of low pathogen diarrhoea in growing pigs – a pilot study

**DOI:** 10.1186/2046-0481-67-24

**Published:** 2014-11-01

**Authors:** Ken Steen Pedersen, Markku Johansen, Øystein Angen, Sven Erik Jorsal, Jens Peter Nielsen, Tim K Jensen, Roberto Guedes, Marie Ståhl, Poul Bækbo

**Affiliations:** Pig Research Centre, Danish Agriculture & Food Council, Axelborg, Axeltorv 3, DK-1609 Copenhagen V, Denmark; National Veterinary Institute, Technical University of Denmark, Bülowsvej 27, 1790 Copenhagen V, Denmark; HERD – Centre for Herd-oriented Education, Research and Development, Department of Large Animal Sciences, University of Copenhagen, Groennegaardsvej 2, DK-1870 Frederiksberg C, Denmark; Department of Veterinary Clinics and Surgery, Veterinary School, Universidade Federal de Minas Gerais, Av. Antônio Carlos 6627, Belo Horizonte, MG 31.270-901 Brazil

**Keywords:** Diagnostic procedures, Low pathogen diarrhoea, Quantitative polymerase chain reaction

## Abstract

**Background:**

The major indication for antibiotic use in Danish pigs is treatment of intestinal diseases post weaning. Clinical decisions on antibiotic batch medication are often based on inspection of diarrhoeic pools on the pen floor. In some of these treated diarrhoea outbreaks, intestinal pathogens can only be demonstrated in a small number of pigs within the treated group (low pathogen diarrhoea). Termination of antibiotic batch medication in herds suffering from such diarrhoea could potentially reduce the consumption of antibiotics in the pig industry. The objective of the present pilot study was to suggest criteria for herd diagnosis of low pathogen diarrhoea in growing pigs.

Data previously collected from 20 Danish herds were used to create a case series of clinical diarrhoea outbreaks normally subjected to antibiotic treatment. In the present study, these diarrhoea outbreaks were classified as low pathogen (<15% of the pigs having bacterial intestinal disease) (n =5 outbreaks) or high pathogen (≥15% of the pigs having bacterial intestinal disease) (n =15 outbreaks). Based on the case series, different diagnostic procedures were explored, and criteria for herd diagnosis of low pathogen diarrhoea were suggested. The effect of sampling variation was explored by simulation.

**Results:**

The diagnostic procedure with the highest combined herd-level sensitivity and specificity was qPCR testing of a pooled sample containing 20 randomly selected faecal samples. The criteria for a positive test result (high pathogen diarrhoea outbreak) were an average of 1.5 diarrhoeic faecal pools on the floor of each pen in the room under investigation and a pathogenic bacterial load ≥35,000 per gram in the faecal pool tested by qPCR. The bacterial load was the sum of *Lawsonia intracellularis*, *Brachyspira pilosicoli* and *Escherichia coli* F4 and F18 bacteria per gram faeces. The herd-diagnostic performance was (herd-level) diagnostic sensitivity =0.99, diagnostic specificity =0.80, positive predictive value =0.94 and negative predictive value =0.96.

**Conclusions:**

The pilot study suggests criteria for herd diagnosis of low pathogen diarrhoea in growing pigs. The suggested criteria should now be evaluated, and the effect of terminating antibiotic batch medication in herds identified as suffering from low pathogen diarrhoea should be explored.

**Electronic supplementary material:**

The online version of this article (doi:10.1186/2046-0481-67-24) contains supplementary material, which is available to authorized users.

## Background

The development of antimicrobial resistance in both animals and humans has led to concern regarding the level of antibiotic consumption in the pig industry [1]. The use of antibiotics is dominated by batch medication, and approximately 35% of all antibiotics used in Danish pigs are used therapeutically for the treatment of intestinal disease [2]. Clinical decisions on antibiotic batch medication are often based on inspection of diarrhoeic pools on the pen floor. *Lawsonia intracellularis* has been considered to be the major intestinal infection causing diarrhoea in growing pigs [3]. However, other intestinal infections exist, including enterotoxinogenic *Escherichia coli, Brachyspira pilosicoli, Brachyspira hyodysenteriae* and *Salmonella* spp. Some diarrhoea outbreaks may be due to other non-infectious factors, since only low pathogen numbers can be demonstrated despite the occurrence of diarrhoea [4, 5].

These diarrhoea outbreaks could be termed low pathogen diarrhoea outbreaks (LP diarrhoea) and potentially do not require antibiotic treatment, since bacterial infections can be demonstrated in no or only a few pigs. Termination of antibiotic treatment regimes in herds suffering from such LP diarrhoea outbreaks could potentially result in a reduction in the amount of antibiotics used for treating diarrhoea, thereby avoiding unnecessary use of antibiotics in pig production. Before general recommendations on non-treatment can be given, two aspects need to be addressed. First, it is necessary to explore whether an accurate herd diagnosis of LP diarrhoea can be obtained in practice by a simple and cheap diagnostic procedure. Second, the implications of terminating antibiotic treatment for animal welfare and productivity must be evaluated.

Disease diagnosis in individual animals relies on collecting clinical, pathological and laboratory diagnostic data as well as choosing the appropriate sample and diagnostic test. Disease diagnosis at the herd level also requires selecting the appropriate number of animals and collection strategy (random versus targeted) based on the expected prevalence of disease. Furthermore, for diagnostic procedures to be used in practice, low laboratory costs, ease of sampling and documentation of the diagnostic value are important.

The objective of the present pilot study was to suggest criteria for herd diagnosis of LP diarrhoea in growing pigs.

## Methods

### Case series of diarrhoea outbreaks

Data previously collected from 20 Danish herds were used to create a case series of clinical diarrhoea outbreaks illustrating diarrhoea outbreaks that are normally subjected to antibiotic treatment in the Danish pig industry. The dataset consisted of 313 euthanised pigs (designated as EUTHA-pigs), 157 of which had diarrhoea and 156 of which did not have diarrhoea. The pigs were, on average, 32 days post-weaning (standard deviation =14 days; range: 12–63 days). At the time of the herd visits where the pigs were selected, the mean within-room prevalence of diarrhoea was 33% (range between herds: 18 - 71%) in the rooms in which the diarrhoea outbreak occurred.

The selection of herds and pigs, the herd examinations, the sample processing and the laboratory investigations have previously been reported [6, 7]. Feed type was recorded as home-mixed/purchased pelleted feed, while days post-weaning and days since the last change in feed were recorded on a continuous scale. A number of additional laboratory investigations of the pigs not previously reported were performed using techniques previously described: colon tissue samples were examined for *B. pilosicoli* by fluorescent in-situ hybridisation (FISH) [8], jejunum content was examined for Rotavirus by ELISA, *Salmonella* spp. and *E. coli* [7], colon content was examined for *Brachyspira* spp [9], and faecal samples were subjected to qPCR testing for *E. coli* F4 and F18 genes [10].

In addition to the 313 euthanised pigs, an additional 480 faecal samples (designated as FAECAL-pigs in the study) were collected from the same diarrhoea outbreaks during the same herd examinations. In each diarrhoea outbreak, these faecal samples were obtained from the rectum of 24 pigs (12 pigs without diarrhoea and 12 with diarrhoea) randomly selected using the previously reported selection process [6]. These faecal samples were subjected to qPCR testing for *L. intracellularis*, *B. pilosicoli* and *E. coli* F4 and F18 genes as previously described [10].

### Classification of diarrhoea as high pathogen or low pathogen outbreaks

Criteria for LP diarrhoea were suggested by the authors and discussed by a panel of five veterinary experts in porcine health. They agreed that antibiotic batch medication should be performed when approximately 15% or more of the pigs within a batch are suffering from a bacterial internal disease. Therefore, an LP diarrhoea outbreak was defined as an outbreak with fewer than 15% of all the pigs (both normal and diarrhoeic pigs) within the outbreak suffering from bacterial intestinal disease when the diagnostic samples were obtained. Based on this definition, each of the 20 diarrhoea outbreaks in the case series was classified as HP or LP diarrhoea.

For each outbreak, the individual EUTHA-pigs were used to calculate the within-outbreak prevalence of pigs (both normal and diarrhoeic pigs) suffering from bacterial intestinal disease at the time of diagnostic sampling.

For the individual EUTHA-pigs, the criteria used for bacterial intestinal disease associated with *L. intracellularis*, *B. pilosicoli* and E*. coli* were as outlined below.

*E. coli*: Demonstration of haemolytic *E. coli* in pure/dominant culture from either faeces or jejunum content.

*L. intracellularis*: Demonstration of *L. intracellularis* in one or more intestinal sections by immunohistochemistry in combination with histological proliferative lesions in the same intestinal sections.

*B. pilosicoli*: Demonstration of *B. pilosicoli* in colon tissue samples by FISH or in faeces/colon intestinal content by culture. In addition to the demonstration of *B. pilosicoli*, there should be a demonstration of dilatation and/or elongation and/or accumulation of cell debris and/or mucus in colon crypts or structures compatible with spirochetes in the lumen of colon crypts.

### Exploring criteria for herd diagnosis

Herd-level diagnostic sensitivity (H_sen_) and specificity (H_spe_), and positive (H_ppv_) and negative (H_npv_) predictive values were calculated for the clinical signs, qPCR test results and co-factors displayed in Table 1. The diarrhoea outbreak classification (high pathogen and low pathogen) was used as the reference standard in the calculations. All the diagnostic procedures that contained diagnostic criteria were measured on a (pseudo) continuous scale (e.g. the number of diarrhoeic pools in pens, the within-outbreak prevalence of qPCR positive pigs, the excretion level in pooled faecal samples, etc.). For these diagnostic criteria, a number of non-parametric receiver operating characteristic analyses (ROC analysis) including ROC curves were performed by calculation of H_sen_, H_spe_, H_ppv_ and H_npv_ for different cut points, e.g. different numbers of diarrhoeic pools in pens. The diagnostic procedures also included combinations of qPCR testing and assessment of the number of diarrhoeic faecal pools in the pens.

**Table 1:** **Characteristics of diarrhoea outbreaks included in cases series**

	Low pathogen diarrhoea outbreaks (n = 5)			High pathogen diarrhoea outbreaks (n = 15)		
	Median	Minimum	Maximum	Median	Minimum	Maximum
Within-outbreak prevalence of bacterial intestinal disease:						
All pigs^a^	0.02	0.00	0.06	0.35	0.16	0.91
Diarrhoeic pigs^a^	0.10	0.00	0.25	0.57	0.17	1.00
Non-diarrhoeic pigs^a^	0.00	0.00	0.00	0.33	0.00	0.86
*Escherichia coli* ^b^	0.00	0.00	0.06	0.15	0.00	0.91
*Lawsonia Intracellularis* ^b^	0.00	0.00	0.02	0.07	0.00	0.55
*Brachyspira pilosicoli* ^b^	0.00	0.00	0.00	0.02	0.00	0.27
Euthanized pigs per batch	16	15	16	16	14	16
Size of batch (number of pigs)	280	210	586	378	184	650
Within-outbreak prevalence of diarrhoea	0.26	0.19	0.32	0.35	0.18	0.71
Within-outbreak prevalence of pigs with sign of intestinal disease^c^	0.41	0.23	0.45	0.29	0.19	0.59
Average diarrhoeic faecal pools in pens	1.8	0.8	4.6	4.0	1.5	9.2
Days post weaning	29	21	42	28	12	63
Days since feed-change	9	0	21	9	2	21
Within-outbreak prevalence of intestinal infections:						
Diarrhoea pigs^d^	0.38	0.17	0.50	0.75	0.17	1.00
Diarrhoea pigs adjusted qPCR^e^	0.14	0.00	0.38	0.67	0.00	0.88
All pigs^d, f^	0.27	0.25	0.69	0.75	0.21	0.94
All pigs adjusted qPCR^e, f^	0.19	0.06	0.20	0.50	0.06	0.71
Faecal load of intestinal infections:						
Mean excretion in diarrheic pigs^g^	352,359	4,150	918,000,000	14,700,000	6,333	2,620,000,000
Mean excretion in all pigs^f, g^	634,348	249,426	490,000,000	7,597,158	4,820	1,480,000,000
Mean excretion in qPCR positive diarrheic pigs^g^	704,721	24,902	2,450,000,000	16,500,000	17,642	2,950,000,000
Mean excretion in qPCR positive all pigs^f, g^	2,537,078	364,322	1,840,000,000	10,100,000	22,333	1,760,000,000

^a^Within outbreak prevalence of pigs with bacterial intestinal disease associated with Escherichia coli, Lawsonia intracellularis and/or Brachyspira pilosicoli (using diarrhoea prevalence as sampling weight to adjust for stratified random sampling).

^b^Within outbreak prevalence of pigs with bacterial intestinal disease associated with the listed bacterium (using diarrhoea prevalence as sampling weight to adjust for stratified random sampling).

^c^Within-outbreak prevalence of pigs having one or more of the following clinical signs: watery diarrhoea, fibrin, blood, mucus or necrotic material in faeces, pale, hairy, and/or unthrifty appearance.

^d^Within-outbreak prevalence of diarrhoeic pigs with detection of *Escherichia coli* F4, F18, *Lawsonia intracellularis* and/or *Brachyspira pilosicoli* by qPCR.

^e^Within-outbreak prevalence of diarrhoeic pigs with detection of Escherichia coli F4, F18, Lawsonia intracellularis and/or Brachyspira pilosicoli by qPCR (Adjusted qPCR indicates that a faecal sample should contain >5.2 log10 copies/g faeces for Escherichia coli F18 or Lawsonia intracellularis in order to be classified as positive for those bacteria).

^f^All pigs indicate that normal and diarrhoeic pigs were equally included in the calculation of the within-outbreak prevalence (i.e. diarrhoea prevalence was not used as sampling weight to adjust for stratified random sampling).

^g^Sum of *Escherichia coli* F4, F18, *Lawsonia intracellularis* and/or *Brachyspira pilosicoli* copies/g faeces.

Clinical and microbiological findings in batches of growing pigs suffering from high pathogen or low pathogen diarrhoea outbreaks from 20 Danish herds where clinical diarrhoea outbreaks were normally subjected to antibiotic treatment.

### Effect of sampling variation

Approximately ten diagnostic procedures representing the highest herd-level sensitivity and specificity were further explored. A computer simulation was performed in order to determine the number of faecal samples necessary to include in each diagnostic procedure in order to obtain valid diagnostic results. For example, should a pooled faecal sample contain five, ten or 20 individual faecal samples? For this task, the qPCR test results from the faecal samples obtained from the FAECAL-pigs were used to address a potential bias caused by the EUTHA-pigs being used both for classification of the reference diarrhoea outbreaks and evaluation of the diagnostic procedures.

The simulations were performed according to the sampling strategy described in each of the diagnostic procedures (Table 1). For each diagnostic procedure, random samples of pigs were selected (drawn by the computer software) from the FAECAL-pig data and classified at herd-level as test-negative (LP diarrhoea outbreak) or test-positive (HP diarrhoea outbreak) according to the criteria in each diagnostic protocol. The qPCR test results from the selected FAECAL-pigs were used. Pooling was simulated as previously described by calculating the mean number of *L. intracellularis*, *B. pilosicoli* and *E. coli* F4 and F18 genes based on the individual test results of the faecal samples contributing to each pool [11]. The selection of pigs from the FAECAL-pig data and classification of the test results in relation to each diarrhoea outbreak were repeated 10,000 times for each diagnostic procedure. Following the simulations, H_sen,_ H_spe,_ H_ppv_ and H_npv_ were calculated for each diagnostic procedure. The reference standard in the calculations was the previously established classification as HP and LP diarrhoea for each diarrhoea outbreak based on the EUTHA-pigs.

All data handling, simulation and statistical analyses were performed using commercial software (Stata/IC, version 12, StataCorp LP, College Station, TX).

### Ethical approval

In the present study no experimental research on animals was performed. The study only involved procedures normally used for routine diagnostics. Danish laws do not require ethical approval for studies not involving different treatment groups or blood testing.

## Results

Laboratory examinations of the euthanised pigs (EUTHA-pigs) demonstrated that *L. intracellularis*, *B. pilosicoli* and E*. coli* were the only bacterial intestinal pathogens.

The within-outbreak prevalence of pigs suffering from bacterial intestinal disease is displayed in Table 1. Data for each of the 20 outbreaks are provided as additional files. Based on the within-outbreak prevalence, five (25%) outbreaks were classified as low pathogen (LP) diarrhoea and 15 (75%) outbreaks were classified as high pathogen (HP) diarrhoea. The LP diarrhoea outbreaks had a within-outbreak prevalence of pigs with bacterial intestinal disease ranging from 0 to 6.4%. The HP diarrhoea outbreaks had a mean within-outbreak prevalence of pigs with bacterial intestinal disease of 41% (range: 16-91%). *E. coli*-associated intestinal disease was demonstrated in 73% (n =11), *L. intracellularis*-associated intestinal disease was demonstrated in 47% (n =7) and *B. pilosicoli*-associated intestinal disease was demonstrated in 47% (n =7) of the diarrhoea outbreaks. Intestinal disease associated with only a single bacterial pathogen was demonstrated in 40% of the outbreaks, while intestinal disease associated with two or three bacterial pathogens was demonstrated in 40% and 20% of the outbreaks, respectively. The within-outbreak prevalence of selected clinical signs, qPCR test results and excretion levels are displayed in Table 1. Data for each of the 20 outbreaks are provided as additional files [see Additional file 1].

H_sen_, H_spe_, H_ppv_ and H_npv_ were calculated for a total of 41 different diagnostic protocols, applying the results from the four different qPCR tests, clinical signs and co-variables displayed in Table 2. The qPCR results included the within-outbreak prevalence of pigs, where one or more of the infections were demonstrated using two different threshold loads, the mean excretion level in faeces for individual qPCR positive pigs and the excretion level in pooled faecal samples. Calculation of H_sen_, H_spe_, H_ppv_ and H_npv_ in the ROC analyses demonstrated that it was difficult to obtain simultaneously high values of both H_sen_ and H_spe._

**Table 2:** **Diagnostic performance of different diagnostic procedures**

	H _sen_*	H _spe_*	H _ppv_*	H _npv_*
**Procedure 1**: 1 of 4 qPCR-tested diarrhoeic pigs should be positive for *L. intracellularis*, *B. pilosicoli*, *E. coli* F4 and/or F18	0.96	0.35	0.82	0.76
**Procedure 2**: 1 of 4 qPCR-tested diarrhoeic pigs should be positive for *L. intracellularis*, *B. pilosicoli*, *E. coli* F4 and/or F18^#^	0.93	0.63	0.88	0.76
**Procedure 3**: 1 of 10 qPCR-tested diarrhoeic pigs should be positive for *L. intracellularis*, *B. pilosicoli*, *E. coli* F4 and/or F18	1.00	0.20	0.79	1.00
**Procedure 4**: 1 of 10 qPCR-tested diarrhoeic pigs should be positive for *L. intracellularis*, *B. pilosicoli*, *E. coli* F4 and/or F18^#^	1.00	0.60	0.88	1.00
**Procedure 5**: 2 of 10 qPCR-tested diarrhoeic pigs should be positive for *L. intracellularis*, *B. pilosicoli*, *E. coli* F4 and/or F18^#^	1.00	0.60	0.88	0.99
**Procedure 6**: Average number of diarrhoeic faecal pools per pen in the room should be ≥1.5, and the sum of *L. intracellularis, B. pilosicoli*,	0.96	0.72	0.91	0.85
*E. coli* F4 and F18 determined by qPCR in a pooled faecal sample containing five samples from diarrhoeic pigs should be ≥6,300 bacteria/g.				
**Procedure 7**: Average number of diarrhoeic faecal pools per pen in the room should be ≥1.5, and the sum of *L. intracellularis, B. pilosicoli*,	1.00	0.66	0.90	1.00
*E. coli* F4 and F18 determined by qPCR in a pooled faecal sample containing 10 samples from diarrhoeic pigs should be ≥6,300 bacteria/g.				
**Procedure 8**: Average number of diarrhoeic faecal pools per pen in the room should be ≥1.5, and the sum of *L. intracellularis, B. pilosicoli*,	0.83	0.86	0.95	0.63
*E. coli* F4 and F18 determined by qPCR in a pooled faecal sample containing five samples from random pigs should be ≥35,000 bacteria/g.				
**Procedure 9**: Average number of diarrhoeic faecal pools per pen in the room should be ≥1.5, and the sum of *L. intracellularis, B. pilosicoli*,	0.94	0.81	0.94	0.82
*E. coli* F4 and F18 determined by qPCR in a pooled faecal sample containing ten samples from random pigs should be ≥35,000 bacteria/g.				
**Procedure 10**: Average number of diarrhoeic faecal pools per pen in the room should be ≥1.5, and the sum of *L. intracellularis, B. pilosicoli*,	0.99	0.80	0.94	0.96
*E. coli* F4 and F18 determined by qPCR in a pooled faecal sample containing 20 samples from random pigs should be ≥35,000 bacteria/g.				
**Procedure 11**: Average number of diarrhoeic faecal pools per pen in the room should be ≥2.5, and 2 of 10 qPCR-tested diarrhoeic pigs should be positive for *L. intracellularis, B. pilosicoli, E. coli* F4 and/or F18^#^	1.00	0.40	0.83	1.00

*H_sen_: Herd-level diagnostic sensitivity; H_spe_: Herd-level specificity; H_ppv_: Herd-level positive predictive value; H_npv_: Herd-level negative predictive value.

^#^The individual qPCR test for *E. coli* F18 and *L. intracellularis* was considered test-positive if a sample contained ≥5.2 log_10_ bacteria/g faeces.

Diagnostic performance determined by computer simulation of 11 different diagnostic procedures used for demonstration of high pathogen diarrhoea outbreaks in growing pigs (all diagnostic procedures use qPCR testing as the only laboratory technique). Diagnostic procedure No. 10 was considered the best.

A total of 11 diagnostic procedures with the highest herd-level sensitivity and specificity were included in the simulation study. A description of the diagnostic procedures and the results for H_sen_, H_spe_, H_ppv_ and H_npv_ are shown in Table 1. The diagnostic procedure with the highest combined values of H_sen_ and H_spe_ was qPCR testing of a single pooled faecal sample, combined with information on the number of diarrhoeic faecal pools in the pens under investigation. The pool had to contain 20 randomly selected faecal samples (approximately half diarrhoea and half normal faeces). The criteria for a positive test result (HP diarrhoea outbreak) were an average of more than 1.5 diarrhoeic faecal pools on the floor of each pen in the room under investigation and a pathogenic bacterial load ≥35,000 per gram in the single pooled faecal sample tested by qPCR. The pathogenic bacterial load was determined by the sum of *L. intracellularis*, *B. pilosicoli* and *E. coli* F4 and F18 bacteria per gram of faeces obtained from the qPCR testing. Using the diagnostic procedure with these criteria gave H_sen_ =0.99, H_spe_ =0.80, H_ppv_ =0.94 and H_npv_ =0.96. The effect of including information on the number of diarrhoeic faecal pools was an increased H_spe_ and H_ppv_. Using an identical diagnostic procedure but without information on diarrhoeic faecal pools gave H_sen_ =0.99, H_spe_ =0.60, H_ppv_ =0.88 and H_npv_ =0.95. For example, the criterion for a positive test result (HP diarrhoea outbreak) was a pathogenic bacterial load ≥35,000 per gram in the single pooled faecal sample tested by qPCR.

## Discussion

The reused dataset applied as the reference standard material demonstrated that 25% of the diarrhoea outbreaks (five of 20 herds) had a very small number of pigs suffering from bacterial intestinal disease. Similar cases of diarrhoea have previously been reported in growing pigs [4, 5]. We suggest using the term low pathogen (LP) diarrhoea for these kinds of diarrhoea outbreaks. LP diarrhoea is a group-level diagnosis, characterised by non-haemorrhagic diarrhoea in approximately 20% of the pigs or more in a group in which known bacterial pathogens can be demonstrated in fewer than 15% of the pigs within the group.

The working hypothesis for the present study was that these LP diarrhoea outbreaks do not require antibiotic treatment and, furthermore, that it would be possible to set criteria for an accurate herd diagnosis of LP diarrhoea that could be applied in veterinary practice. This could potentially result in a reduction in the consumption of antibiotics used for treating diarrhoea. Today, these diarrhoea outbreaks will, under normal Danish practical conditions, result in antibiotic batch medication, and therefore a diagnostic procedure should be able to identify the non-infectious outbreaks (high herd-level diagnostic specificity) in order to reduce antibiotic consumption. On the other hand, it could result in severe production losses if antibiotic treatment regimes were terminated in herds that were actually suffering from HP diarrhoea. Therefore, the diagnostic procedure needed to result in diagnostic test results with a high probability (high herd-level negative predictive value) that the outbreak would be truly LP diarrhoea when a negative test result was obtained. The results of the study demonstrated that it was difficult to select a diagnostic procedure and criteria that would provide acceptable levels of both herd-level sensitivity and specificity. The diagnostic procedure that performed best had H_spe_ =0.80 and H_npv_ =0.96. This implies that it will be possible to identify 80% of the LP diarrhoea outbreaks, with a negligible risk of the HP diarrhoea outbreaks being falsely classified. In this diagnostic procedure, the sum of the pathogenic bacterial loads was used without taking the individual bacterial species into consideration. This procedure was considered acceptable, since the majority of the diarrhoea outbreaks involved different combinations of *L. intracellularis-*, *E. coli-* and *B. pilosicoli-*associated intestinal disease.

The acceptable levels of H_spe_ =0.80 and H_npv_ =0.96 were only achieved by combining clinical information (diarrhoeic faecal pools in pens) with qPCR testing. The diagnostic procedure includes counting the total number of diarrhoeic faecal pools in all pens within the room under investigation and dividing that number by the number of pens. Such a clinical procedure can potentially be subject to inter- and/or intra-observer variation. This aspect needs to be addressed in order to determine the effect on repeatability for the diagnostic procedure. Omitting the clinical information and relying solely on the qPCR testing implies that it will only be possible to identify 60% of the LP diarrhoea outbreaks. However, the risk of falsely classifying a HP diarrhoea outbreak as LP diarrhoea remains negligible.

Testing pooled faecal samples will reduce diagnostic costs, and, interestingly, pooling of faecal samples provided better diagnostic performance compared with testing of individual samples. Furthermore, pooling a larger number of faecal samples increased sensitivity without a major reduction in specificity, resulting in an increased negative predictive value for herd diagnosis. The most likely biological explanation for this is the relatively high prevalence of pigs not having a bacterial intestinal disease (with or without diarrhoea) in most of the diarrhoea outbreaks. This implies that increasing the number of pigs represented in a pooled sample will increase the probability that a faecal sample from a pig suffering from bacterial intestinal disease is included.

Two of the LP diarrhoea outbreaks contained high levels of pathogenic bacteria tested by qPCR. In both cases, presence of *E. coli* F18 genes in faeces was the primary cause of the high excretion level (data not shown). This indicates that the level of F18 in faeces is not always correlated to intestinal disease. Potential explanations are that some pigs do not have F18 receptors in the intestines or that F18 genes are also present in non-pathogenic *E. coli.* Including one or more qPCR tests for other *E. coli* virulence genes could be a way to increase the diagnostic performance in relation to *E. coli.* Further, because the qPCR results from the four qPCR tests were summed together, inclusion of other *E. coli* virulence genes could potentially influence the diagnostic performance of the suggested diagnostic strategy in herds experiencing mixed infections.

In the current study, haemolysis was used as a virulence marker for *E. coli*. It would have been preferable if an examination for virulence genes had been performed to confirm the pathogenic classification used. A previous study under Danish conditions has demonstrated that haemolysis will provide an acceptable typing method for identification of pathogenic *E. coli* compared with molecular typing methods [12]. Based on the result of this previous study, the most likely bias resulting from the use of haemolysis is that some non-haemolytic *E. coli* has been falsely classified as non-pathogenic. This could potentially result in the misclassification of HP diarrhoea as low pathogen diarrhoea outbreaks, leading to an overestimation of diagnostic sensitivity and underestimation of diagnostic specificity for the suggested diagnostic procedure in the current study.

The present study should be considered a pilot study. The suggested diagnostic criteria should now be evaluated under field conditions to address whether an accurate herd diagnosis of LP diarrhoea can be obtained using the criteria. However, currently the suggested diagnostic criteria could potentially be used as a first step in investigating outbreaks of diarrhoea in specific herds. If an outbreak in a herd is determined to be potentially LP diarrhoea, the antibiotic batch medication should be terminated in one or two batches to evaluate whether the terminated antibiotic strategy will result in any negative clinical effect. On the other hand, if a diarrhoea outbreak in a herd is determined to be HP diarrhoea, other laboratory investigations may follow to perform a more in-depth assessment of the infections involved, including assessment of antibiotic resistance.

Diarrhoea classified as LP diarrhoea could potentially progress into an infectious diarrhoea outbreak at a later time point post weaning when treatment has been terminated. Therefore, a diagnostic investigation should be repeated later if new cases of diarrhoea develop in the same pigs. In this way, the diagnostic procedure could potentially be used to determine the correct time point for antibiotic batch medication in herds suffering from diarrhoea.

The suggested diagnostic procedure should only be used in relation to diagnostic investigations of diarrhoea, because the study was performed on data illustrating diarrhoea outbreaks that are normally subjected to antibiotic treatment.

Another important aspect of antibiotic treatment is the occurrence of subclinical intestinal disease without any signs of diarrhoea. It is possible that a similar diagnostic approach using qPCR testing of pooled faecal samples could also be useful in determining the correct time point for batch treatment of subclinical cases of intestinal disease. A working hypothesis for future research could be that repeated qPCR testing of pooled faecal samples within batches of pigs would eliminate the need for clinical monitoring of diarrhoea and/or diarrhoeic faecal pools in the pens.

One aspect that needs investigation is the reproducibility within a herd. In swine practice, it is common to perform diagnostic investigations in one group of sick pigs and extrapolate the diagnostic results to other groups or upcoming batches of pigs within the same herd. However, the disease dynamics within a herd may change between batches. This could potentially also apply to the occurrence of LP diarrhoea outbreaks, and this aspect needs further investigation.

In the present study, an LP diarrhoea outbreak was defined as an outbreak with fewer than 15% of all the pigs (normal and diarrhoeic pigs) within the outbreak suffering from bacterial intestinal disease when the diagnostic samples were obtained. Both the normal and the diarrhoeic pigs were included in the prevalence calculations, because batch medication subjects all pigs within a room to antibiotic treatment at the same time, including healthy, subclinically and clinically affected pigs. The threshold of 15% may be subject to discussion based on concerns related to the development of antimicrobial resistance, animal welfare issues and economic aspects. Furthermore, it could be relevant in future research to address how changing the threshold affects the performance of the diagnostic procedure.

## Conclusions

The present study should be considered a pilot study. The results suggest diagnostic criteria for herd diagnosis of LP diarrhoea in growing pigs. The suggested criteria should now be further evaluated under field conditions, including an investigation of the effect of terminating antibiotic batch medication in herds with a herd diagnosis of LP diarrhoea.

## Electronic supplementary material

Additional file 1: **Clinical and microbiological data for a case series of 20 diarrhoea outbreaks.** (XLSX 23 KB)

## References

[1] Collignon P, Powers JH, Chiller TM, Aidara-Kane A, Aarestrup FM (2009). World Health Organization ranking of antimicrobials according to their importance in human medicine: a critical step for developing risk management strategies for the use of antimicrobials in food production animals. Clin Infect Dis.

[2] Hybschmann GK, Ersboll AK, Vigre H, Baadsgaard NP, Houe H (2011). Herd-level risk factors for antimicrobial demanding gastrointestinal diseases in Danish herds with finisher pigs: a register-based study. Prev Vet Med.

[3] Jensen HM (2006). Health management with reduced antibiotic use - experiences of a Danish pig vet. Anim Biotechnol.

[4] Chase-Topping ME, Gunn G, Strachan WD, Edwards SA, Smith WJ, Hillman K, Stefopoulou SN, Thomson JR (2007). Epidemiology of porcine non-specific colitis on Scottish farms. Vet J.

[5] Pedersen KS, Kristensen CS, Nielsen JP (2012). Demonstration of non-specific colitis and increased crypt depth in colon of weaned pigs with diarrhea. Vet Q.

[6] Pedersen KS, Stahl M, Guedes RM, Angen O, Nielsen JP, Jensen TK (2012). Association between faecal load of lawsonia intracellularis and pathological findings of proliferative enteropathy in pigs with diarrhoea. BMC Vet Res.

[7] Pedersen KS, Stege H, Jensen TK, Guedes R, Stahl M, Nielsen JP, Hjulsager C, Larsen LE, Angen O (2013). Diagnostic performance of fecal quantitative real-time polymerase chain reaction for detection of Lawsonia intracellularis-associated proliferative enteropathy in nursery pigs. J Vet Diagn Invest.

[8] Boye M, Jensen TK, Moller K, Leser TD, Jorsal SE (1998). Specific detection of the genus Serpulina, S. hyodysenteriae and S. pilosicoliin porcine intestines by fluorescent rRNA in situ hybridization. Mol Cell Probes.

[9] Moller K, Jensen TK, Jorsal SE, Leser TD, Carstensen B (1998). Detection of Lawsonia intracellularis, Serpulina hyodysenteriae, weakly beta-haemolytic intestinal spirochaetes, Salmonella enterica, and haemolytic Escherichia coli from swine herds with and without diarrhoea among growing pigs. Vet Microbiol.

[10] Stahl M, Kokotovic B, Hjulsager CK, Breum SO, Angen O (2011). The use of quantitative PCR for identification and quantification of Brachyspira pilosicoli, Lawsonia intracellularis and Escherichia coli fimbrial types F4 and F18 in pig feces. Vet Microbiol.

[11] Pedersen KS, Johansen M, Jorsal SE, Nielsen JP, Bækbo P, Angen Ø (2014). Pooling of porcine fecal samples for quantification of Lawsonia intracellularis by real-time polymerase chain reaction. J Vet Diagn Invest.

[12] Frydendahl K (2002). Prevalence of serogroups and virulence genes in *Escherichia coli* associated with postweaning diarrhoea and edema disease in pigs and a comparison of diagnostic approaches. Vet Microbiol.

